# Marine and brackish *Luticola* D.G.Mann (Bacillariophyta) species from the Java Sea and South China Sea coasts with the description of three new species

**DOI:** 10.3897/phytokeys.183.71049

**Published:** 2021-10-22

**Authors:** Mateusz Rybak, Andrzej Witkowski, Łukasz Peszek, John P. Kociolek, Yenny Risjani, Duc Hung Nguyen, Jinpeng Zhang, Yunianta, Van Duy Nguyen, Romain Gastineau, Thi Thuy Duong, Philippe Rosa, Vona Meleder

**Affiliations:** 1 Department of Agroecology and Forest Utilization, Institute of Agricultural Sciences, Land Management and Environmental Protection, University of Rzeszów, ul. Ćwiklińskiej 1a, 35-601 Rzeszów, Poland; 2 University of Szczecin, Institute of Marine and Environmental Sciences, Mickiewicza 16A, 70-383 Szczecin, Poland; 3 Natural Sciences Education and Research Centre, University of Szczecin, Mickiewicza 16a, 70-383 Szczecin, Poland; 4 Museum of Natural History and Department of Ecology and Evolutionary Biology University of Colorado, Boulder, Colorado, 80309 USA; 5 Faculty of Fisheries and Marine Sciences, Brawijaya University, Jl. Veteran, 65145 Malang, Indonesia; 6 Faculty of Natural Sciences Pedagogy, Saigon University, Ho Chi Minh City, Vietnam; 7 Guangzhou Marine Geological Survey, China Geological Survey/Key Laboratory of Marine Mineral Resources, Ministry of Natural Resources, 188 Guanghai Rd., Guangzhou 510760, China; 8 Faculty of Agricultural Technology, Brawijaya University, Jl. Veteran, 65145 Malang, Indonesia; 9 Institute of Environmental Technology, Vietnam Academy of Sciences and Technology, 18 Hoang Quoc Viet Road, Cau Giay, Hanoi, Vietnam; 10 Université de Nantes, EA 2160 Mer – Molécules – Santé 2, Rue de la Houssinière, 44322 Nantes, France

**Keywords:** Brackish waters, *Luticola* genus, marine tropical coasts, morphology, offshore aquaculture

## Abstract

In this study, samples were collected from the Java Sea coasts, from the South China Sea in Hainan Island coasts and Quảng Yên region and Rú Chá mangrove near Hue in Central Vietnam. In studied samples a total of eight *Luticola* species have been observed. Three of the taxa studied are described herein as species new to science – *Luticolaorientalis***sp. nov.**, *L.cribriareolata***sp. nov.** and *L.halongiana***sp. nov.** Under light microscopy (LM) *L.orientalis***sp. nov.** and *L.cribriareolata***sp. nov.** are similar with rhombic-lanceolate to rhombic/ elliptic-lanceolate to elliptic valve shapes and narrowly rounded apices. Both species can be easily distinguished by stria density (higher density in *L.orientalis*). Under SEM*L.cribriareolata* is characterized by cribrate areola occlusions, a character thus far observed only in three established species. The remaining species of the whole genus known thus far are characterized by hymenate areola. Similar morphology *Luticola* species have been observed from tropical mangrove forests from Madagascar but they all can be easily distinguished based on the lack of grooves in the central area. The third species – *L.halongiana***sp. nov.** has rhombic-elliptic to rhombic-lanceolate valves with broadly rounded to slightly protracted apices in larger specimens. This species has a relatively broad central area. Also unique among brackish-water *Luticola* is the small, rounded stigma positioned almost midway between the valve center and valve margin. In the habitats from which the new species are described we also identified five established *Luticola* taxa including, *L.belawanensis*, *L.celebesica*, *L.inserata*, *L.seposita* and *L.tropica*. For those species we provide detailed SEM characteristics of valve ultrastructure, as well as the range of environmental conditions and geographic distribution within the study area.

## Introduction

The genus *Luticola* was established in Round et al. (1990). It shows great variability in size and shape of the valve, as well as in the types of environment in which they may occur. In a monograph on the genus *Luticola*, (Levkov et al. 2013) presented about 200 species, 93 of which they described as new. The taxonomic revision and update on established taxa, and description of a wealth of new ones performed by the above authors, are considered to be starting points for further taxonomic research on *Luticola*. In the following papers descriptions or appropriate transfers of almost 50 taxa from various parts of the world have been published (Guiry and Guiry 2021). Most of the new taxa descriptions and new combinations are concerned with species found in Asia (Glushchenko and Kulikovskiy 2015; Kale et al. 2017; Glushchenko et al. 2017; Liu et al. 2017; Lokhande et al. 2020). Numerous new taxa have also been described from the Antarctic region (Zidarova et al. 2014; Kohler et al. 2015; Chattová et al. 2017; Kochman-Kędziora et al. 2020) as well as from South America (Bąk et al. 2017; Bustos et al. 2017; Straube et al. 2017; Da Silva-Lehmkuhl et al. 2019; Simonato et al. 2020), from Europe (Levkov et al. 2017; Coste et al. 2019; Hindáková and Noga 2021) and from Madagascar (Bąk et al. 2019). And, most recently, three *Luticola* species new to science were described from the cave entrance of one of the most remote islands in the World Ocean, the Rapa Nui (i.e. Easter Island, Chile) (Peszek et al. 2021).

Material for this study was collected from various microhabitats from coastal regions surrounding the Java Sea coasts (north coast of Java Island) and from the South China Sea in Hainan Island coasts (S China), Quang Yen, Quang Ninh province (NE Vietnam) and Ru Cha mangrove, Thua Thien Hue province (Central Vietnam). Collections were derived from a wide range of salinities, from a range of brackish water sites up to a fully marine site, and included various biofilms from tidal mudflats, oyster shells, rocks and hydrotechnical constructions that turned out to host abundant and sometimes even dominant populations of *Luticola* species. Most of the species observed in our samples are well-known from the tropical ocean coasts across the globe and are known to prefer brackish water environments (Levkov et al. 2013). Brackish water and marine habitats are unusual for *Luticola* species as most of them either inhabit freshwaters or can be found in various kinds of terrestrial habitats including soils (e.g. Levkov et al. 2013, 2017; Zidarova et al. 2014; Kochman-Kędziora et al. 2020). Until now only a few species have been reported from brackish environments and, except the generic type species *L.mutica* which can be abundant in European estuaries (Levkov et al. 2013; Lange-Bertalot et al. 2017), almost all of them occur in the tropics (Levkov et al. 2013). Some, including e.g. *L.tropica* Levkov, Metzeltin & Pavlov, are widely distributed in tropical estuaries (Fernandes et al. 1990; Navarro and Lobban 2009; Levkov et al. 2013; Straube et al. 2017; Glushchenko et al. 2017) whereas the others occur in more restricted areas like in waters from SE Asia to the coast of Australia (*L.belawanensis* Levkov & Metzeltin, *L.inserata* (Hustedt) D.G.Mann, *L.lacertosa* (Hustedt) D.G.Mann, *L.novaeguineaensis* (Tempère) Levkov, Metzeltin & Pavlov) (Foged 1978; Levkov et al. 2013; Glushchenko et al. 2017), to Madagascar (*L.madagascarensis* M.Bąk, Kryk & Peszek and *L.nosybeana* Kryk, M.Bąk & Peszek in Bąk et al. 2019) and to Galapagos islands (*L.galapagoensis* Witkowski, Bąk, Kociolek, Lange-Bertalot & Seddon and *L.darwinii* Witkowski, Bąk, Kociolek, Lange-Bertalot & Seddon in Bąk et al. 2017). It is worth mentioning that the morphologically similar genus *Luticolopsis* Levkov, Metzeltin & Pavlov, which is monotypic (*L.vietnamica* Levkov, Metzeltin & Pavlov), is also found inhabiting brackish water habitats (Levkov et al. 2013).

The aim of this paper is to provide a description of three new species – *Luticolaorientalis* M.Rybak, Peszek, JP.Zhang & Witkowski sp. nov., *Luticolacribriareolata* Witkowski, M.Rybak, Risjani & Yunianta sp. nov. and *L.halongiana* M.Rybak, Witkowski, H-D.Nguyen & D-V.Nguyen sp. nov. We also provide for the first time detailed characteristics of valve ultrastructure and supplementing of knowledge of the following *Luticola* species: *L.belawanensis* Levkov & Metzeltin and *L.inserata* (Husted) D.G.Mann, *L.seposita* (Hustedt) D.G.Mann and *L.tropica* Levkov, Metzeltin & Pavlov. Based on published sources, the geographic distribution of the established *Luticola* species is provided. These taxa seem to comprise a group of brackish-water to fully marine species confined to tropical coasts, primarily mangroves and tidal flats, but also biofilms on rock surfaces, oyster shells and hydrotechnical constructions.

## Material and methods

### Sampling sites (Fig. 1)

#### Site 1 – The Java Sea, East Java north coast (E Java, N coast); 07°46'42"S, 113°16'34"E

The sampling area was located on the north coast of eastern part of Java Island bordered by the southern part of the Java Sea. In contrast to the south coast of East Java, the northern part of Java is less bright, and has lower light levels penetrating the water column due to, in some places, turbid waters heavily loaded with sediment. Measured environmental parameters according to Risjani et al. 2021 are presented in Table 1. For this study we used samples with accession number SZCZ 27006 and SZCZ 27007, both of which originate from the Probolinggo coastal zone. The habitats sampled involved periphyton from a plastic pier and from the boulders protecting the coastal zone in Probolinggo at Pantai Bentar. Material was collected by Y. Risjani and Yunianta on March 1^st^ 2020.

#### Site 2 – NW South China Sea, Hainan Island; 18°35'2"N, 110°10'31"E

**Figure 1.**
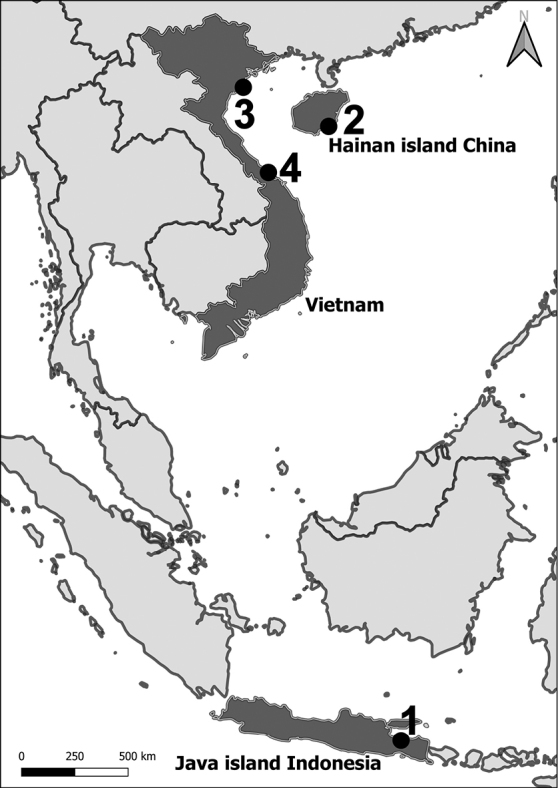
Map showing location of sampling sites: 1–The Java Sea, East Java north coast, 2–NW South China Sea, Hainan Island, China 3–W South China Sea, Quang Ninh province, NE Vietnam, 4–Rú Chá mangrove, Central Vietnam.

The sampling area was located on the coast of Hainan Island, in the NW South China Sea (Fig. 1). This island has numerous bays (e.g. Yangpu Bay, Sanya Bay) and provides suitable habitats for rich diatom assemblages of sandy beaches, rocks, mangroves and coral reefs with numerous hydrotechnical constructions (Hainan Provincial Local History Office 2020; Hainan Provincial Bureau of Statistics 2020). The coastal sea water temperature around Hainan Island in winter, at Haikou is 18.7 °C, while in Sanya in the south it increases to 22 °C. The annual difference in sea water temperature oscillates between 7 and 11 °C (Hainan Provincial Local History Office 2020; Hainan Meteorological Service 2020). The average annual salinity of the surface seawater along the coast of Hainan Island is 32.64. The salinity extremes are as high as 36.0 psu and 36.2 psu in Dongfang and Yinggehai respectively (Hainan Provincial Local History Office 2020). Water transparency ranges between 0.5 and 20 meters with eastern and southern coastal areas highly transparent, while the western and northern regions are less transparent. Abundant populations of Luticola spp. were recorded in the sample with accession number SZCZ27176 collected by A. Witkowski and J.P. Zhang on April 20^th^ 2015. It was a microbial mat developed on a pier at Fenjiezhou Island. Results of the measured environmental parameters are in Table 1.

**Table 1.** Samples characteristics and physicochemical parameters of water of studied sites (– means no data available).

Sampling site	Site 1: The Java Sea, East Java north coast	Site 2: NW South China Sea, Hainan Island	Site 3:W South China Sea, Quáng Yên, NE Vietnam	Site 4: Rú Chá mangrove, Central Vietnam
Sample number	SZCZ 27006 SZCZ 27007	SZCZ27176	SZCZ26472	SZCZ26505
Sample type	periphyton from a plastic pier and boulders	microbial mat developed on a pier	periphyton from oyster shells from aquaculture	sediments from mangrove area
Water temperature [°C]	30.0–32.6	24.9	27–32	29.6
pH	7.7–8.3	8.2	5.6–6.6	6.5
Salinity [psu]	27.7–30.4	32.8	22.0–23.0	7.0
Conductivity [µS/cm]	–	–	34 700–36 500	–
Dissolved oxygen [%]	–	–	–	5.9
Dissolved oxygen [mg/L]	3.5–6.6	–	–	0.45

#### Site 3 – W South China Sea, Quáng Yên, Quáng Ninh province, NE Vietnam; 20°13'20"N, 106°32'14"E

Quáng Yên is one of the coastal towns of Quáng Ninh province that is located in the northern part of Vietnam, and the biggest oyster (*Ostreaedulis* Linnaeus, 1758) aquaculture area of the Ha Long Bay. Quáng Yen has a climate characteristic of the tropical monsoon with cold winters. Total rainfall amounts to ca. 500–700 mm. Quáng Yên is considered an area sensitive to the impacts of climate change regarding mangrove forests and biodiversity. Periphyton from oyster shells from offshore aquaculture facility, by V. Méléder, P. Rosa and T.T. Duong on October 28^th^ 2018. Accession number SZCZ26472. Water parameters measured in situ are in Table 1.

#### Site 4 – W South China Sea, Rú Chá mangrove, Thua Thien Hue province, Central Vietnam; 16°33'28"N, 107°36'41"E

Rú Chá mangrove functions as an ecotone between the mainland and the lagoon. With an overall area of about 50 to 100 hectares, the core species of the area of more than 5 hectares is *Excoecariaagallocha* L. The mangrove flora in Rú Chá has 27 species (10 true mangrove species and 17 mangrove associated species. In 2014, an assessment of surface water and sediment of the Rú Chá mangrove showed that the surface water had a high concentration of total nitrogen (3.4 mg L^-1^) and total phosphorus (0.3 mg L^-1^). The sediments were saline, strongly acidic, frequently waterlogged and rich in organic matter (Ha et al. 2015). A sample with access number SZCZ26505 was collected by V. Méléder and P. Rosa on November 11^th^ 2018 from the mangrove area. Water parameters measured *in situ* are presented in Table 1.

### Diatom analysis

Diatom samples were collected using tooth brush to detach the periphyton from solid substrate (pier, boulders) and with a plastic tube pressed into the sediment in case of soft substrate (microbial mat, sediment). Diatom samples were cleaned by boiling with 30% hydrogen peroxide (H_2_O_2_) for a few hours. Cleaned diatom material was pipetted on to coverslips and dried, and then mounted on glass slides using Naphrax mounting medium (Brunel Microscopes Ltd, Wiltshire, U.K.). Light microscopy (LM) observations were made with a Zeiss Axio Imager A2 (Carl Zeiss, Jena, Germany) using a × 100 Plan Apochromatic oil immersion objective (NA 1.46) equipped with Differential Interference Contrast (DIC). Diatom images were captured with a Zeiss AxioCam ICc5 camera (Jena, Germany). For scanning electron microscope (SEM) examination, a few drops of cleaned material were put onto Whatman Nuclepore polycarbonate membrane filters (Fisher Scientific, Schwerte, Germany). Once dried, the membranes were mounted on to aluminum stubs and coated with 20 nm of gold using a turbo-pumped Quorum Q 150T ES coater. SEM observations were performed at the University of Rzeszów, using a Hitachi SEM SU8010. The diatom terminology follows: Round et al. (1990) and Levkov et al. (2013).

## Results

### Descriptions of new *Luticola* species

#### Phylum: Bacillariophyta Haeckel

**Class: Bacillariophyceae Haeckel**

**Subclass: Bacillariophycidae D.G.Mann**

**Order: Naviculales Bessey**

**Family: Diadesmidaceae D.G.Mann**

##### Genus: *Luticola* D.G.Mann in Round et al. (1990)

###### Luticola orientalis

Taxon classificationPlantaeNaviculalesDiadesmidaceae

M.Rybak, Peszek, JP.Zhang & Witkowski sp. nov.

2BFAB715-BE63-5579-8515-C71BF5813762

Figures 2A –AH, 3

####### Description LM.

Valves rhombic-lanceolate to rhombic in smaller specimens with narrowly rounded apices. Valves 9.5–22.1 μm in length, 5.4–8.5 μm in width (n = 30). Raphe filiform, axial area narrow and linear expanding into rectangular, narrow central area, stigma side of the central area bordered by 2–3 areolae, on side opposite stigma bordered by 1–2 areolae. Stigma located close to valve margin. Transapical striae easily distinguishable with LM, radiate throughout, 18–22 in 10 μm.

####### Description SEM.

Valve surface flat, the transition between valve face and the mantle abrupt marked with a stripe of hyaline silica. Axial area narrow becoming broader toward the valve middle, expanding into the rectangular central area. Externally raphe filiform and straight, distally strongly hooked in the same direction on valve apices, proximal raphe endings close to each other, simple and clearly bent towards the primary valve side (opposite the stigma). Valve mantle with a single row of elliptical areolae. Internally raphe branches straight, with proximal ends simple and relatively distant, terminating at the apices as small, indistinct helictoglossae. Transapical striae composed of 4–6 rounded or slightly transapically elongated areolae, often becoming smaller close to valve margin, internally occluded by hymenes. Areola occlusions positioned at the internal valve surface. Externally elongated stigma positioned close to valve margin of the valve secondary side. Internal stigma opening with large-lipped structure. Internally longitudinal channel visible on face and mantle conjunction, with relatively large silica flap on site opposite to stigma opening. Longitudinal channel covered by hymen similar to those occluding areola.

**Figure 3.**
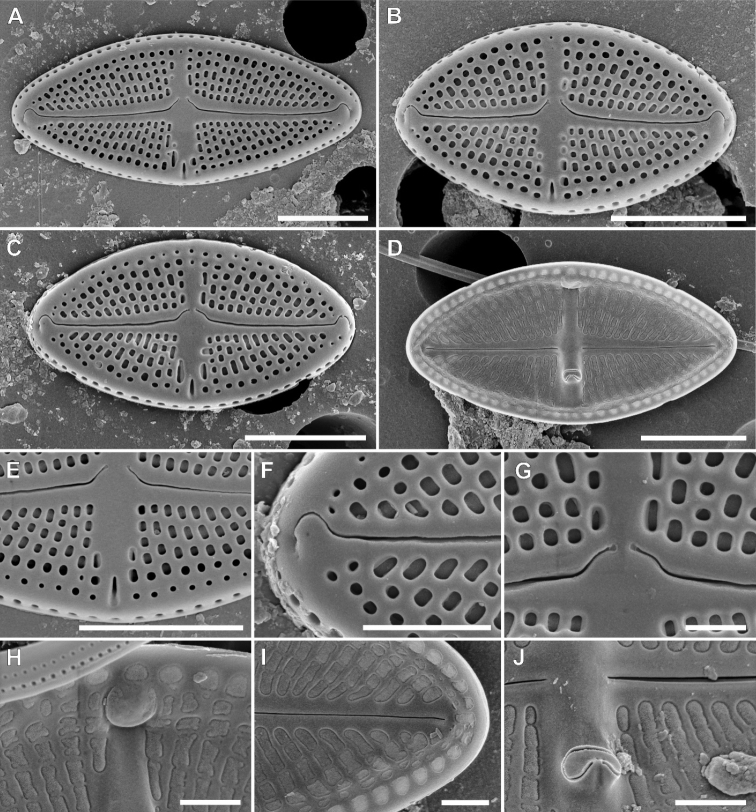
SEM micrographs of *Luticolaorientalis* M.Rybak, Peszek, JP.Zhang & Witkowski sp. nov. External valve view (**A–C; E-G**), Internal valve view (**D; H–J**). Detailed view of showing external view of stigma opening **E** distal **F** and proximal **G** raphe endings. Detailed close-ups showing internal silica flap on longitudinal channel **H** distal raphe endings **I** Detailed view of proximal raphe endings and stigma opening (J). Scale bars: 5 µm (**A–D**), 1 µm (**E, G, I, J**), 2 µm (**F, H**).

####### Holotype.

**Figure 2.**
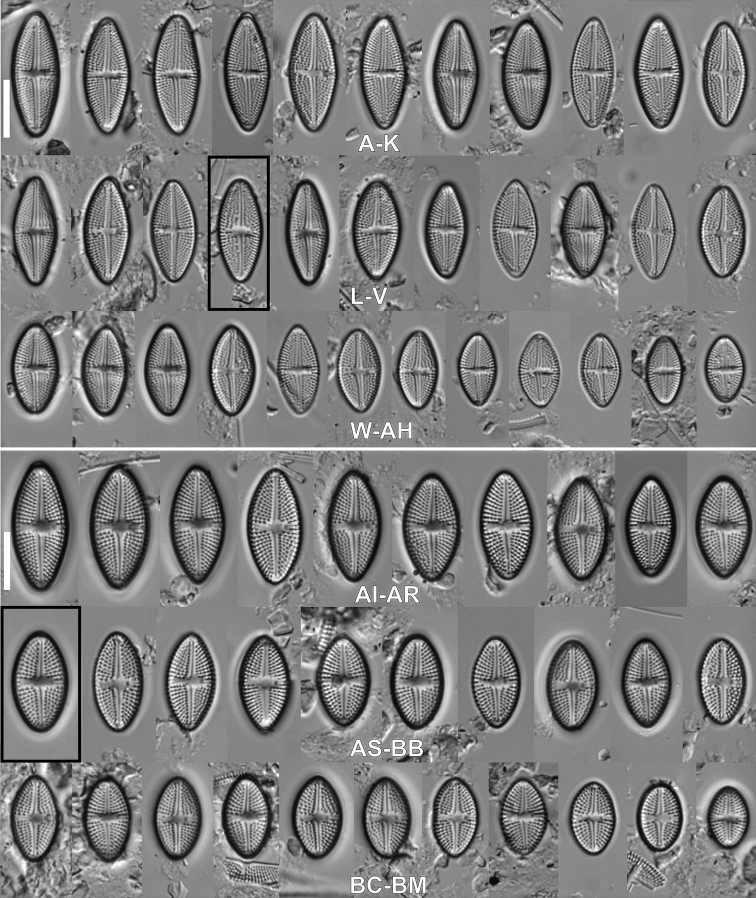
LM micrographs of size diminution series of *Luticolaorientalis* M.Rybak, Peszek, JP.Zhang & Witkowski sp. nov. (**A–AH**) and *Luticolacribriareolata* M.Rybak, Witkowski, Risjani & Yunianta sp. nov. (**AI–BM**). Holotype specimen of *Luticolaorientalis* sp. nov. – black frame (**O**). Holotype specimen of *Luticolacribriareolata* sp. nov. – black frame (**AS**). Scale bar: 10 µm.

Slide SZCZ27007 stored in A. Witkowski Diatom Collection of the Institute of Marine and Environmental Sciences, University of Szczecin, holotype specimen is Fig. 2O.

####### Isotype.

Slide no. 2018/425 and unmounted material with the same number at the University of Rzeszów, Poland.

####### Type locality.

Indonesia. Java Island: Pantai Bentar in Probolinggo at North coast, a periphyton from a boulder, 07°46'41"S, 113°16'34"E, *leg. Y. Risjani*, *Yunianta* and *A. Witkowski 1^st^ March 2020*.

####### Etymology.

The name refers to the geographical location – east (lat. *orientalis* – eastern).

####### Distribution.

Abundant in holotype sample SZCZ27007, and in periphyton from the plastic pier at Pantai Bentar in Probolinggo, and was also present in sample SZCZ27006 very close to the holotype habitat. The new species was also observed in an epilithic sample from Fenjiezhou Island at the coast of Hainan Island, NW South China Sea in sample SZCZ27176, and from the Xuân Thúy Mangrove in NE Vietnam where it was found in the biofilm from wild oysters, sample SZCZ26472.

####### Taxonomic comment.

Valve shape of *Luticolaorientalis* sp. nov. is similar to *Luticolacribriareolata* sp. nov., however, the former species can be distinguished by stria density, which are finer than in *L.cribriareolata* sp. nov. *Luticolaorientalis* sp. nov. is also similar in terms of valve outline to *L.nosybeana* and *L.madagascarensis* from Nosy Be Island, however, the former species has simple proximal raphe endings without any grooves (Table 2) which are distinct in both Madagascar species (Bąk et al. 2019).

###### Luticola cribriareolata

Taxon classificationPlantaeNaviculalesDiadesmidaceae

M.Rybak, Witkowski, Risjani & Yunianta sp. nov.

BBB52AFC-1401-5C05-9CB7-83FCB52BC205

####### Description LM.

Valves elliptic-lanceolate to elliptic with rounded apices. Valves 9.8–28.3 μm in length, 6–11.6 μm in width (n = 30). Raphe filiform, axial area narrow at apices becoming broader towards valve middle part, expanding into asymmetrical central area, broader opposite the stigma and bordered by 2–3 areolae. Stigma present close to valve margin. Transapical striae easily distinguishable with LM, radiate throughout, 14–16 in 10 μm.

####### Description SEM.

Valve surface flat with the transition to the mantle abrupt and marked with distinct hyaline stripe. Axial area narrow becoming broader toward the valve middle, expanding into the rectangular central area. Raphe filiform and straight, external proximal raphe endings close to each other, clearly bent to the valve primary side and associated with irregular in shape shallow grooves expanded in the direction opposite the stigma. External raphe distal ends strongly hooked on valve face and terminating in an indistinct groove in central area, at apical part of mantle. Valve mantle steep with a single row of oblong areolae. Girdle composed of a few copulae each with two rows of small circular pores. Internally, raphe branches straight, with proximal endings slightly bent. Internally, raphe terminates in a small helictoglossae. Transapical striae composed of 2–5 large areolae. Areolae on both valve face and valve mantle are deeply embedded, and occluded with reticulated cribra positioned on the inner valve surface. Within central area ghost areolae are often observed, oblong to strongly elongated in shape. Elongated stigma positioned close to margin of the valve primary side. Externally, stigma small and slightly elongated. Internal stigma opening with large-lipped structure. Internally, longitudinal channel visible, with small silica flap on site opposite to stigma. Internally, areolae and longitudinal channel occluded with irregular hymenate structure.

####### Holotype.

Slide SZCZ27007 stored in A. Witkowski Diatom Collection of the Institute of Marine and Environmental Sciences, University of Szczecin, represented here by Fig. 2AS.

####### Isotype.

Slide no. 2018/425 and unmounted material with the same number at the University of Rzeszów, Poland.

####### Type locality.

Indonesia. Java Island: Pantai Bentar in Probolinggo on the north coast, a periphyton from a boulder, 07°46'41"S, 113°16'34"E, *leg. Y. Risjani*, *Yunianta* and *A. Witkowski*, *1^st^ March 2020*.

**Figure 4.**
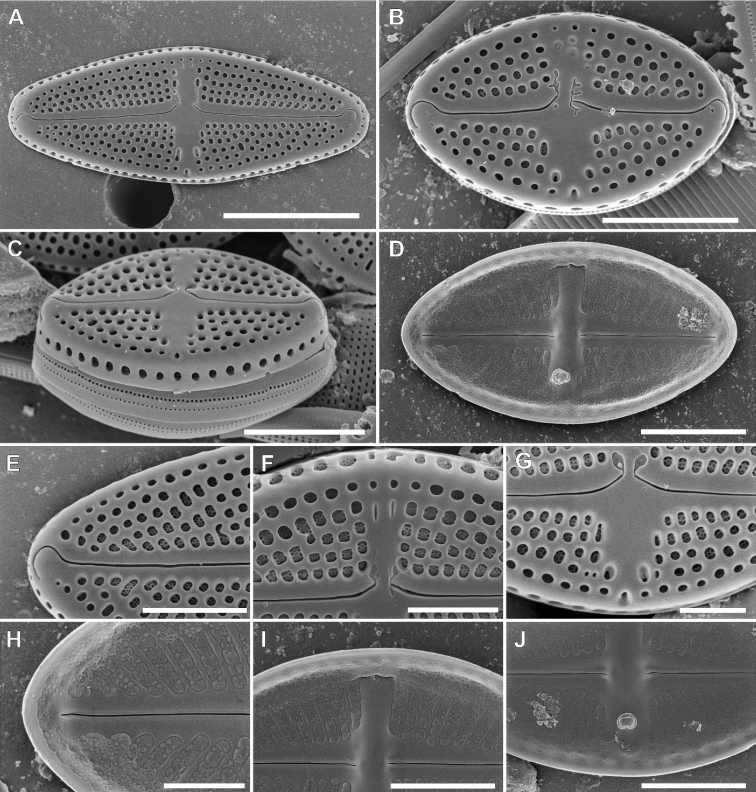
SEM pictures of *Luticolacribriareolata* M.Rybak, Witkowski, Risjani & Yunianta sp. nov. External valve view (**A–C, E–G**), External view with cribrated mantle areolae **C** internal valve view (**D, H–J**). External details of distal raphe ending and areolae **E** proximal raphe endings with shallow grooves, and ghost areolae **F** proximal raphe endings with shallow grooves and stigma opening **G** internal details of raphe branch with distal raphe end and irregular hymenate structure **H** proximal raphe endings and longitudinal channel **I** proximal raphe endings and stigma opening **J**. Scale bars: 10 µm (**A, C, D**), 5 µm (**B**), 4 µm (**E, I, J**), 3 µm (**F**), 2 µm (**G, H**).

####### Etymology.

The species name is derived from its areola occlusions which are in the shape of reticulated cribra, hence the stem “cribr-” of the word “cribrum” is left, the connecting vowel “-i-” and “areolata” are added = cribriareolata.

####### Distribution.

Observed thus far from the holotype sample SZCZ27007, and in periphyton from the plastic pier at Pantai Bentar in Probolinggo and in sample SZCZ27006 very close to the holotype habitat.

####### Taxonomic comment.

*Luticolacribriareolata* has valve shape similar to *Luticolaorientalis* sp. nov., however, the two species can be easily distinguished based on the stria density, which is coarser in *L.cribriareolata*. *Luticolacribriareolata* sp. nov. is also similar in terms of valve outline to *L.nosybeana* and *L.madagascarensis* described from Nosy Be Island from NW Madagascar. The newly described species has simple proximal raphe endings, whereas both *L.madagascarensis* and *L.nosybeana* have external proximal raphe endings with distinct grooves (Bąk et al. 2019) (Table 2).

###### Luticola halongiana

Taxon classificationPlantaeNaviculalesDiadesmidaceae

Witkowski, M.Rybak, H-D.Nguyen & D-V.Nguyen sp. nov.

9F289AD4-A885-5E3E-A510-E17A4D0BF91D

Figure 5

####### Description LM.

Valves rhombic-eliptic to rhombic-lanceolate with broadly rounded to slightly protracted apices in larger specimens, 9.9–22.1 μm in length and 5.4–7.7 μm in width (n = 30). Raphe filiform, slightly bent, axial area narrow, expanding into rectangular to bow-tie shaped central area bordered by 3–4 areolae. Stigma round, present near the valve margin. Transapical striae radiate throughout, 20–24 in 10 μm.

####### Description SEM.

Valve face flat, the transition from valve face to the mantle abrupt, marked with distinct hyaline stripe. Axial area narrow linear, slightly broadened towards the valve middle, expanding into the bow-tie shaped central area with slit like opening of stigma bordered by 3–4 small, rounded areolae. Raphe filiform and straight. Raphe branches very slightly bent with external proximal raphe endings strongly deflected to the valve primary side (opposite to stigma) with small, rounded grooves on the stigma-bearing side. External distal raphe ends slightly hooked and terminate on the apex valve mantle. The valve mantle bearing one row of oblong areola. Internally, raphe straight, with proximal endings slightly bent, and distal raphe endings terminating in small helictoglossae. Transapical striae composed by 3–4(5) round to slightly elongate areolae. Internally, areolae covered by hymen forming continuous strip. Internal stigma opening with circular lipped structure. Internally, longitudal channel visible, with small silica flap on site opposite to stigma.

**Table 2.** Morphological characteristics of all *Luticola* taxa listed here with comparisons to most similar brackish taxa based on literature data. Data marked with an asterisk (*) are obtained from photomicrographs.

	Size [μm] Length/Width	Striae [in 10 μm]	Areolae characteristic	Proximal raphe endings	Distal raphe endings	Distribution	References
***L.orientalis* sp. nov.**	9.5–22.1/5.4–8.5	18–22	4–6 per striae, round or slightly elongated	slightly deflected, close to each other	hooked	Java, Hainan Island, Vietnam	this study
***L.cribriareolata* sp. nov.**	9.8–28.3/6.0–11.6	14–16	3–5 per striae, with deeply positioned cribrum	slightly deflected with long irregular thread-like grooves	hooked	Java	this study
***L.halongiana* sp. nov.**	9.9–22.1/5.4–7.7	20–24	3–4(5) per striae, round or slightly elongated	strongly deflected with small rounded groove	hooked	Vietnam, Java	this study
***L.belawanensis***	8.4–19.0/6.1–9.0	18–21	3–4(5) per striae mainly slightly elongated	bent with small C-shaped or irregular grooves	hooked	Vietnam	this study
	15.5–27.0/15.5–27	18–20	3–4 per striae	Deflected	–	Sumatra	Levkov et al. 2013
	9–29/5–10	18–19	3–5 per striae*	–	–	Vietnam	Glushchenko et al. 2017
***L.celebesica***	10.6–27.1/7.3–13.1	17–19	4–6 per striae	Deflected	hooked	Vietnam	this study
	11.5–39.0/11.5–39.0	18–21	(4)5–6 per striae	deflected	hooked	Sulawesi	Levkov et al. 2013
***L.nosybeana***	9–27/6.0–10.5	20–24	4–5 per striae, round to elliptic	with irregular “insect-antennae-like” or “butterfly-like” grooves	hooked	Madagascar	Bąk et al. 2019
***L.madagascarensis***	13.0–22.5/6.0–7.5	20–24	3–4 per striae, round to elliptic or slit-like	with L-shaped grooves	hooked	Madagascar	Bąk et al. 2019
***L.inserata***	12.2–33.5/8.2–14.0	15–20	4–5 per striae, round to elongated with small spines on margin	bent with irregular thread-like grooves	hooked	Vietnam	this study
	23–28/12	18	–	–	–	Sumatra	Hustedt 1955
	18–28/10.0–13.5	16–19	5–6 per striae, round to elongated	deflected	hooked	Indonesia, Australia	Levkov et al. 2013
	15–25/9–12	20	4–6 per striae, round to elongated*	–	–	Vietnam	Glushchenko et al. 2017
***L.seposita***	16.8–24.4/9.5–12.4	14–17	4–5 per striae, round to slightly elongated areolae	bent, with small C-shaped grooves	hooked	Hainan Island	this study
	23/11	16–18	–	bent	bent slightly S-shaped	Sulawesi	Hustedt 1942
	18–24/10–12	18–21	4–5 per striae, transapically elongated	Hook-shaped	hooked	–	Levkov et al. 2013
***L.tropica***	11.8–21.2/7.5–11.1	17–20	4–5 per striae	clearly bent with long irregular thread-like grooves	hooked	Hainan Island, Vietnam	this study
	12–22/7–9	20	–	–	–	South Africa	Cholnoky 1960
	15.5–24.0/8–11–5	20–24	4–5 per striae, round to transversally elongated	Bent and expanded into central pores	hooked	–	Levkov et al. 2013
	11–24/7–9	20–24	4–5 per striae*	–	–	Vietnam	Glushchenko et al. 2017
	8.8–19.8/6.3–10.3	16–18	4–5 per striae, round or slightly elongated*	slightly deflected with long irregular thread-like grooves*	hooked*	Brazil	Straube et al. 2017

**Figure 5.**
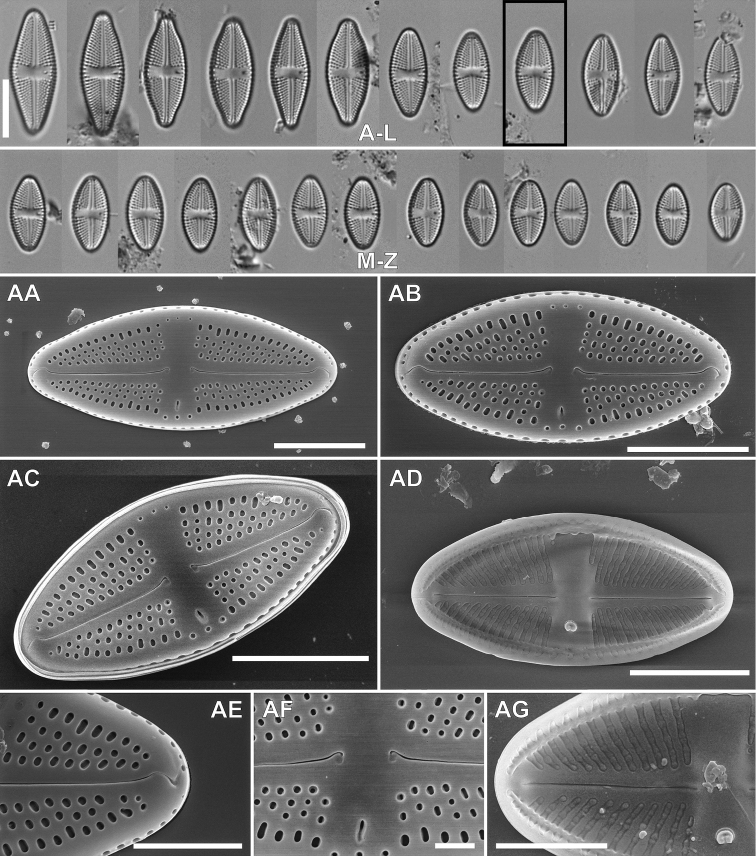
LM micrographs of *Luticolahalongiana* Witkowski, M.Rybak, H-D.Nguyen & D-V.Nguyen sp. nov. in size diminution series (**A–Z**). Holotype specimen – black frame **I** external view of frustule (**AA–AC**). Internal view of frustule (**AD**). Detailed external close-ups showing distal raphe ending (**AE**), proximal raphe endings with small rounded grooves and opening of stigma (**AF**). Detailed internal close-up showing distal and proximal raphe endings, hymen structure and stigma opening (**AG**). Scale bars: 10 µm (**A-Z**), 5 µm (**AA, AB, AC, AD**), 3 µm (**AE, AG**), 1 µm (**AF**).

####### Holotype.

slide SZCZ26472 stored in A. Witkowski Diatom Collection of the Institute of Marine and Environmental Sciences, University of Szczecin, represented here by Fig. 5I

####### Isotype.

Slide no. 2018/425 and unmounted material with the same number at the University of Rzeszów, Poland.

####### Type locality.

NE Vietnam: W South China Sea, Quáng Yên, in Halong region, oyster offshore aquaculture, 20°54'1"N, 106°54'17"E, *leg. Vona Meleder* and *Philipp Rosa*, *10^th^ October 2018*.

####### Etymology.

The specific epithet refers to the type location, Ha Long, NE Vietnam.

####### Distribution.

Species occur rarely, observed thus far at the type locality Quáng Yên in biofilm on shells, and on the north coast of Java in Indonesia, from periphyton from the plastic pier (slide SZCZ27006).

####### Taxonomic comment.

*Luticolahalongiana* sp. nov. has a unique set of characters and it is difficult to point out any similar established species. The only exception is the position of the stigma, which is located almost in the middle between the valve center and valve margin. This makes it similar to *L.madagascarensis*, however, the latter species has external proximal raphe endings with long and distinct grooves on a side opposite the stigma; these grooves are indistinct in *L.halongiana*. Also, *Luticolamutica* (Kützing) D.G.Mann shows some similarities to *L.halongiana* sp. nov. but it can be easily distinguished based on the narrower central area. Also *L.mutica* has areolae containing cribrum which is not present in described species.

Morphological characteristic of recently established *Luticola* taxa observed during this study.

###### Luticola belawanensis

Taxon classificationPlantaeNaviculalesDiadesmidaceae

Levkov & Metzeltin

43A7FD73-D005-5DEB-974C-FE33A503447F

Figure 6

####### Description LM.

Valves elliptic to elliptic-lanceolate with rounded apices. Valve length 8.4–19 μm, breadth 6.1–9.0 μm, with easily distinguishable radiate striae (18–21 in 10 μm) (n = 15). Axial area lanceolate. Central area asymmetrical, with wider side opposite the stigma, bordered on each margin by a row of areolae. Stigma elongated, located close to valve margin.

####### Description SEM.

Valve face flat, raphe filiform and straight, distally strongly hooked on valve face at the apices. Proximal raphe endings clearly bent to the side opposite the stigma with small C-shaped or irregular grooves evident. Internally, raphe straight, proximal endings only slightly bent, whereas distal raphe endings terminate in small helictoglossae. Transapical striae composed of 4–5 round to slightly elongate areolae. Single row of areolae occurs also on valve mantle. Internally, areolae occluded with hymen forming continuous strips. External opening of stigma slit-like, positioned close to valve margin but separated by a single areola. Internal stigma opening with large-lipped structure positioned mid-way between valve margin and valve center. Internally, longitudinal channel present along the valve margin, with small silica flap on side opposite the stigma.

**Figure 6.**
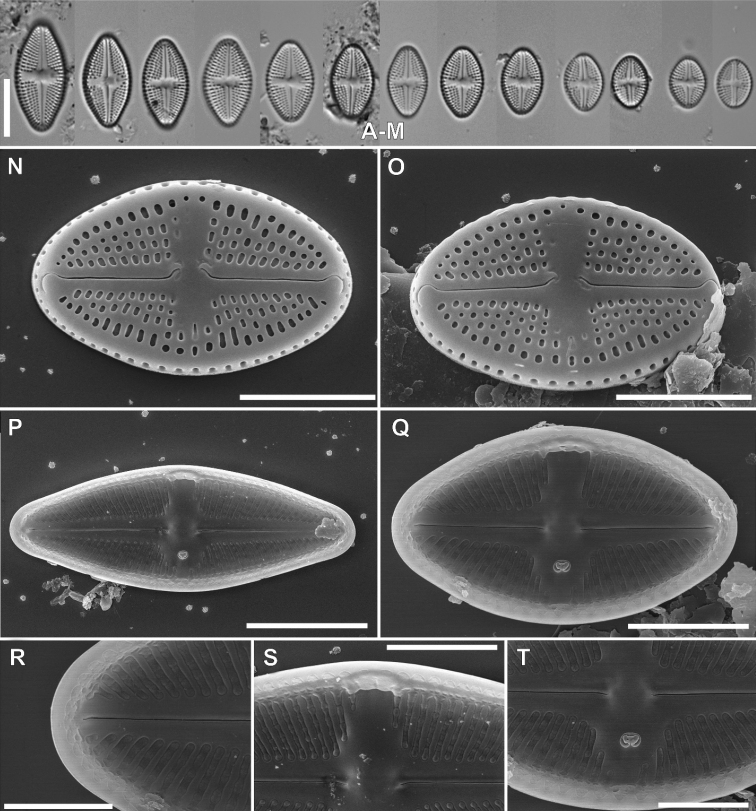
LM pictures of *Luticolabelawanensis* Levkov & Metzeltin in size diminution series (**A–M**). External view of frustule (**N, O**). Internal view of frustule (**P, Q**). Internal details of: raphe branch with distal raphe end and hymen structure **R** proximal raphe endings and longitudinal channel **S** proximal raphe endings and stigma opening **T**. Scale bars: 10 µm (**A-M, P**), 5 µm (**N, O, Q, S**), 3 µm (**R, T**).

####### Distribution.

Occurred rarely only in sample SZCZ26472 from Western South China Sea, Quáng Yên, in Ha Long region of NE Vietnam, collected from oyster shells in offshore aquaculture area.

###### Luticola celebesica

Taxon classificationPlantaeNaviculalesDiadesmidaceae

Levkov, Metzeltin & Pavlov

F62AF6E3-6906-5365-AC41-1D24EF50020F

Figure 7

####### Description LM.

Valves elliptic to rhombic-elliptic with rounded apices, 10.6–27.1 μm in length, 7.3–13.1 μm in width (n = 9). Axial area broad, clearly expanded near central area, asymmetrical bordered by shortened striae, composed of 2–3 areolae while on opposite site of a single areola. Raphe branches straight with hooked distal raphe endings and proximal endings deflected to site opposite to stigma. Transapical striae easily distinguishable, radiate, 17–19 in 10 μm. Slit-shaped stigma located close to valve margin.

**Figure 7.**
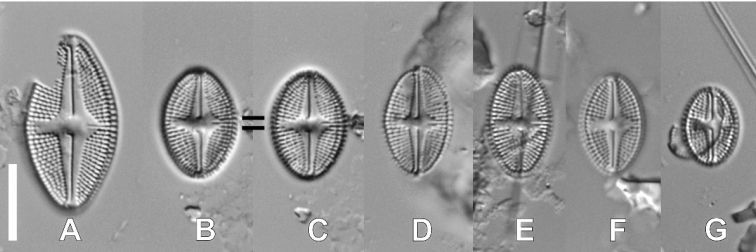
LM micrographs of *Luticolacelebesica* Levkov, Metzeltin & Pavlov in size diminution series (**A–G**). Scale bar: 10 µm.

####### Distribution.

This is a very rare species, observed only in sample SZCZ26505 from Rú Chá Mangrove mud flat in Hue, the western South China Sea coast, Central Vietnam. Due to the rare occurrence of this species, a detailed description of valve ultrastructure was impossible up until the present.

###### Luticola inserata

Taxon classificationPlantaeNaviculalesDiadesmidaceae

(Hustedt) D.G.Mann

2D953ADA-039A-58A3-A7C8-4099BC9D9D90

Figures 8 , 9

####### Description LM.

Valves lanceolate-elliptical to broadly elliptic with weakly undulated margins with rounded, rostrate to capitate apices, 12.2–33.5 μm in length, 8.2–14.0 μm in width (n = 20). Axial area narrow, gradually broadening towards valve center, central area rectangular, asymmetrical, bordered by two or three shortened striae with slit-like stigma located close to valve margin. Transapical striae radiate, becoming strongly radiate toward apices, 15–20 in 10 μm. Copulae open.

####### Description SEM.

Valve face flat, raphe branches filiform and straight. External proximal raphe endings clearly bent to the site opposite the stigma with irregularly-shaped grooves expanded opposite the stigma. External distal raphe endings terminate on apices, strongly hooked. Internally, raphe branches straight, only proximal endings slightly bent, distal raphe endings terminating in small helictoglossae. Transapical striae composed of 4–5 round to elongate areolae. Single row of elongate areolae occurs also on valve mantle. Both areolae on mantle and valve face with small spines on edges. Internally, areolae covered with hymen forming continuous strips. Ghost areolae rarely present within central area. External opening of stigma small and rounded, positioned very close to valve margin. Internal stigma opening with large-lipped structure positioned mid-way between valve margin and valve center. Internally, longitudinal channel present along the valve margin, with small silica flap on side opposite the stigma.

**Figure 8.**
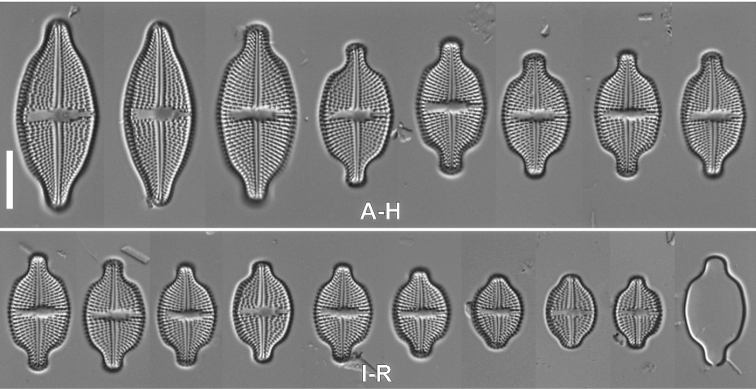
LM micrographs of *Luticolainserata* (Hustedt) D.G.Mann in size diminution series (**A–R**). Scale bar: 10 µm.

**Figure 9.**
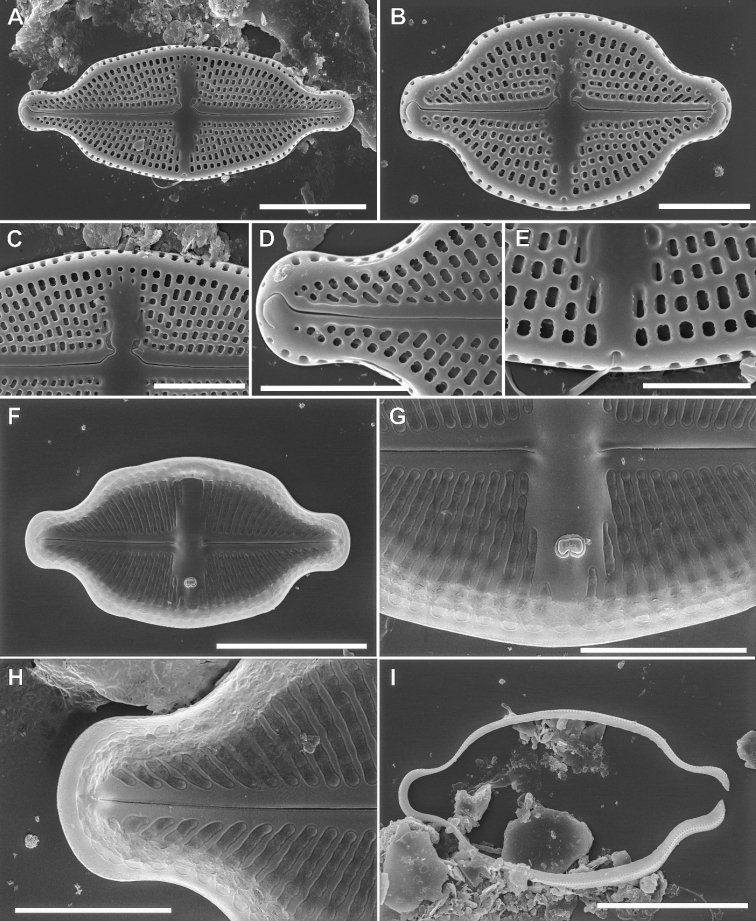
SEM micrographs of *Luticolainserata* (Hustedt) D.G.Mann. External view of valve (**A, B**). Detailed external views showing proximal raphe endings with irregular shallow grooves **C** distal raphe ending, areolae **D** stigma opening **E** internal view of valve **F** detailed internal view showing proximal raphe endings and stigma opening **G** and distal raphe ending and hymenate structure **H** open copulae **I**. Scale bars: 10 µm (**A, F, I**), 5 µm (**B–D, G**), 3 µm (**E**), 4 µm (**H**).

####### Distribution.

This species was observed only in sample SZCZ26472 from Western South China Sea, Quáng Yên, in Halong region of NE Vietnam, shell scrape from oysters in offshore aquaculture area.

###### Luticola seposita

Taxon classificationPlantaeNaviculalesDiadesmidaceae

(Hustedt) D.G.Mann

91ACAEBF-78A1-53DF-BB64-536E34640F4B

Figures 10 , 11

####### Description LM.

Valves linear-elliptic to elliptic with weakly undulate margins, with rounded, rostrate to capitate apices, 16.8–24.4 μm in length, 9.5–12.4 μm in width (n = 20). Axial area narrowly-lanceolate. Central area bordered by two or three shortened striae with slit-like stigma positioned close to the valve margin. Transapical striae radiate becoming strongly radiate toward apices, 14–17 in 10 μm.

####### Description SEM.

Valve face flat, raphe filiform and straight, distally strongly hooked at the apices. External proximal raphe endings bent to side opposite the stigma with small C-shaped grooves. Internally, raphe straight, only proximal endings slightly bent, with distal raphe endings terminating in small helictoglossae. Transapical striae composed of 4–5 round to slightly elongated areolae. Single row of areolae present also on valve mantle. Internally, areolae occluded with hymen forming continuous strips. External elongate ghost areolae present within central area. External opening of stigma slightly elongate and positioned close to valve margin. Internally, as a large-lipped structure positioned mid-way between valve margin and valve center. Internally, longitudinal channel is present along the valve margin, with small siliceous flap on side opposite to stigma.

**Figure 10.**
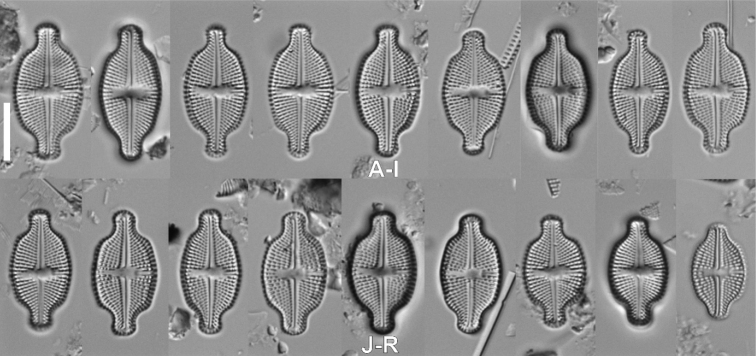
LM micrographs of *Luticolaseposita* (Hustedt) D.G.Mann in size diminution series (**A–R**). Scale bar: 10 µm.

**Figure 11.**
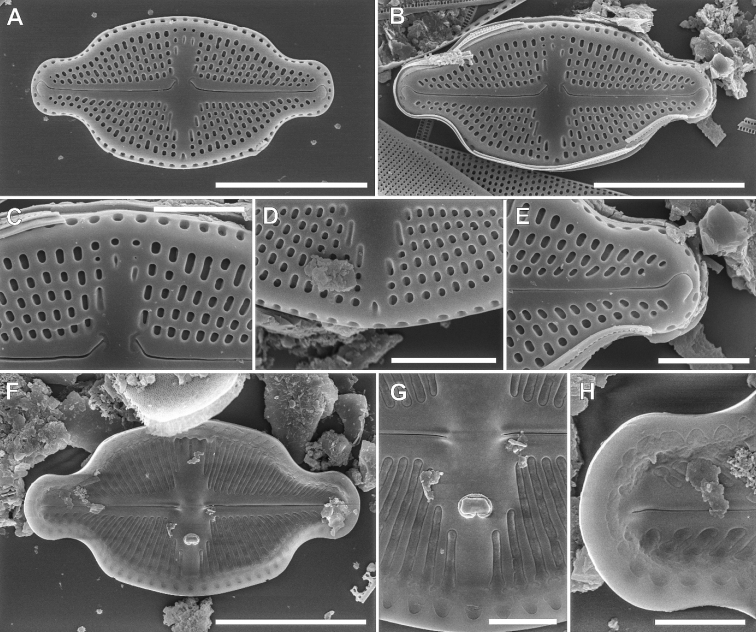
SEM micrographs of *Luticolaseposita* (Hustedt) D.G.Mann. External view of valve (**A, B**). Detailed external views showing proximal raphe endings with C-shaped grooves **C** stigma opening **D** and distal raphe ending **E** internal view of valve **F** detailed internal views showing proximal raphe endings, stigma opening and hymenate structure **G** and distal raphe ending **H**. Scale bars: 10 µm (**A, B, F**), 3 µm (**C, E**), 4 µm (**D**), 2 µm (**G, H**).

####### Distribution.

This species was observed only in epilithic sample from a sampling site called Fenjiezhou Island located on the coast of Hainan Island, NW South China Sea (China) in sample SZCZ27176.

###### Luticola tropica

Taxon classificationPlantaeNaviculalesDiadesmidaceae

Levkov, Metzeltin & Pavlov

AED72F98-4605-52B1-AFF1-5084C68A6B90

Figure 12

####### Description LM.

Valves elliptic-lanceolate with triundulate margins with rostrate and broadly rounded apices, 11.8–21.2 μm in length, 7.5–11.1 μm in width (n = 25). Axial area narrow linear, slightly broadening towards valve middle, expanding into rectangular central area bordered on each margin by 2–4 shortened striae. Transapical striae clearly punctate, radiate becoming strongly radiate toward apices, 17–20 in 10 μm. Stigma slightly elongated, close to the valve margin.

####### Description SEM.

Valve face flat, raphe filiform and straight, distally strongly hooked at the apices. External proximal raphe endings strongly bent to the side opposite the stigma, expanding into thread-like grooves that are variable in shape. Internally, raphe straight, only proximal endings slightly bent, distal raphe endings terminate in small helictoglossae. Transapical striae composed of 4–5 round to slightly elongate areolae. Single row of areolae also occurs on the valve mantle. Internally, areolae occluded with hymen forming continuous strips. A few ghost areolae present within central area. Slightly elongate stigma positioned close to the valve margin. Internal stigma opening with large-lipped structure, located midway between raphe endings and valve margin. Internally, longitudinal channel present along the valve margin, with small siliceous flap on side opposite the stigma.

**Figure 12.**
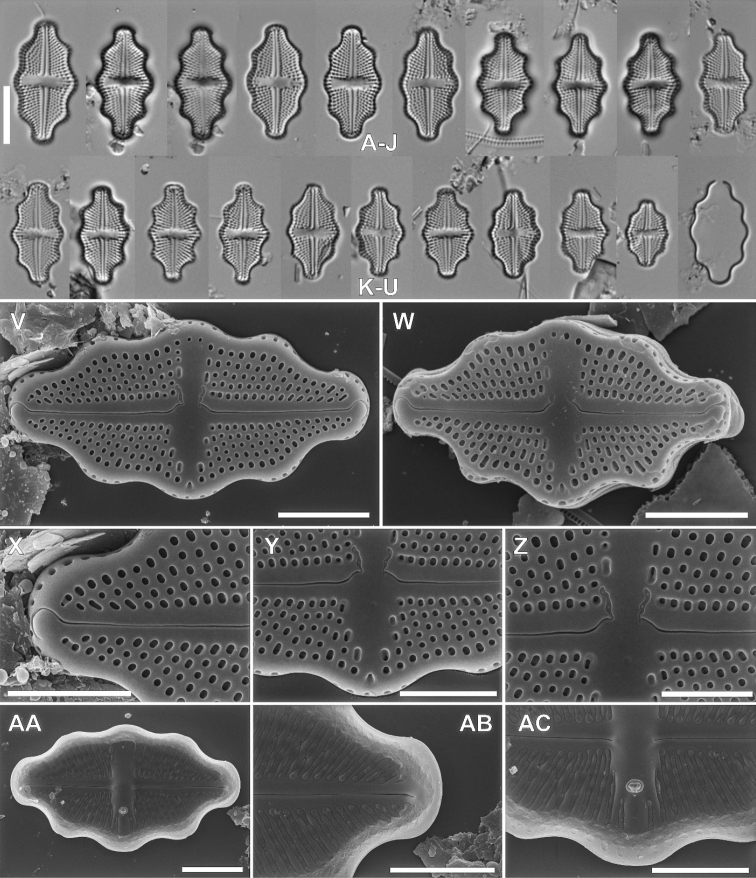
LM micrographs of *Luticolatropica* Levkov, Metzeltin & Pavlov in size diminution series (**A–T**) and isolated open copula **U** external view of valve (**V, W**) Detailed external views showing distal raphe ending **X** stigma opening **Y** proximal raphe endings with thread-like grooves (**Y, Z**) and areolae structure (**X–Z**). Internal view of valve **AA** detailed internal close-ups showing distal raphe ending **AB** proximal raphe endings and stigma opening **AC**. Scale bars: 10 µm (**A–U**), 5 µm (**V, W, AA**), 4 µm (**X, Y, AB, AC**), 3 µm (**Z**).

####### Distribution.

This species was abundant in epilithic sample from a sampling site called Fenjiezhou Island located on the coast of Hainan Island, NW South China Sea (China) in sample SZCZ27176, and in Xuân Thúy Mangrove in NE Vietnam, as biofilm from wild oysters, sample SZCZ26472.

## Discussion

### Brackish and marine water *Luticola*

The genus *Luticola* D.G.Mann contains species with various ecological preferences. However, most of the research on this genus concerns species inhabiting terrestrial and freshwater environments, while the brackish and marine species are still poorly studied. Likewise, poorly known is their geographic distribution and autecology, except the generitype of the genus i.e. *Luticolamutica*, a species widely distributed in estuaries and brackish-water basins of the Northern Hemisphere (e.g. Hofmann et al. 2011; Levkov et al. 2013; Lange-Bertalot et al. 2017).

For the coasts studied to date, *Luticola* species seem to be inhabitants of mudflats and part of various kinds of biofilms related to human activity (oyster shells from offshore aquaculture, hydrotechnical constructions). These habitats have negative impacts on environmental conditions, which seem to be interconnected at least in the north coast of Java and Hainan Island (S China). The existing environmental data suggest that the North Java coast which abounds with *Luticola* spp. is affected by a strong human impact of densely populated coastal area. Likewise, the Vietnamese and Hainan coasts we sampled are well known to be densely populated regions. All these findings related to brackish water *Luticola* species from SE Asia are confirmed by the autecology of *L.mutica* distributed in human impacted rivers (Lange-Bertalot 1979) and estuaries in Europe (Hofmann et al. 2011; Levkov et al. 2013).

Levkov et al. (2013) selected a few *Luticola* taxa confined to tropical brackish-water and marine habitats, but the information on their morphology was mostly only on LM data and the occurrence represents only a few findings. Included in this group were e.g. *L.belawanensis*, *L.inserata*, *L.celebesica* and *L.tropica*. Interestingly, all these species were described from Indonesia and, in particular, from Sumatra Island coastal region either by Hustedt (1942, 1955) and included in Naviculae section Punctatae Cleve or by Levkov et al. (2013). Taxa described by Hustedt were later transferred in *Luticola* by D.G. Mann in Round et al. (1990). Interestingly for the taxa described by Levkov et al. (2013) the holotypes were designated from the slides studied by Hustedt (1942, 1955) although some of these taxa have the pantropical geographic distribution. An example of this pattern can be seen in *L.tropica*. Our LM and SEM observations revealed that the investigated samples from coastal waters of Hainan Island (China), Vietnam and Java coasts show a high *Luticola* diversity in terms of species and high relative abundance. In addition to the established taxa reported herein, we have also observed three taxa new to science. Compared with other regions of the world, coastal areas of SE Asia have high diversity of *Luticola* species. Indeed, several *Luticola* species have been described from brackish-waters of marine coasts of the Nosy Be Island in NW Madagascar (Bąk et al. 2019) and from Laguna Diabla in Isabela Island of Galapagos Archipelago (Bąk et al. 2017). This makes together four species (two from Nosy Be and two from Galapagos) and according to present information the number of established brackish-water and marine *Luticola* with our novel taxa slightly exceeds a dozen (Levkov et al. 2013; Bąk et al. 2017, 2019, this study). However, as shown in our recent study on tropical *Luticola* from marine coasts of China, Indonesia and Vietnam, the potential for discoveries of new species is high if the appropriate habitats are sampled. With several hundred samples from the above coasts we sampled only those enriched in organic matter e.g. tidal flat, biofilm revealed significant content (even dominance) of *Luticola* spp. (Risjani et al. 2021). The highest relative abundance of *Luticola* were observed in highly populated coasts of North Java (Probolinggo area) and in Hainan Island. Whereas in Probolinggo the dominant taxa were *L.cribriareolata* and *L.orientalis*, in Hainan Island these were *L.seposita* and *L.tropica*. Interestingly, the third novel species *L.halongiana* was observed in biofilm on oyster shells from an offshore aquaculture area. It is a well-known fact that *L.goeppertiana* and *L.mutica* are tolerant to high and moderate loads of organic contents in freshwaters and in coastal marine waters (Hofmann et al. 2011). Results of our study seem to conform such high tolerance abilities, at least in the case of *L.orientalis* and *L.cribriareolata*, which are dominant in turbid waters of North Java and Hainan Island.

Two of the newly described species – *L.orientalis* sp. nov. and *L.cribriareolata* sp. nov. show a high similarity to each other in the valve shape. However, they can be easily distinguished based on the stria density. Also valve ultrastructure as resolved with SEM allows for the easy separation of these species (Table 2). These two taxa are similar in their valve outline to two species described from NW Madagascar (Nosy Be): *L.nosybeana* and *L.madagascarensis*. *Luticolaorientalis* sp. nov., despite having overlapping length and width dimensions, can be easily distinguished from both Madagascar species based on denser areolae per stria. Also *L.orientalis* sp. nov. has a much narrower central area than both above mentioned taxa. The major distinguishing characters of the new *Luticola* species versus Madagascar taxa are simple proximal raphe endings without any grooves, which are present both in *L.madagascarensis* and *L.nosybeana* (Bąk et al. 2019), (Table 2). Despite the similar valve outline and presence of grooves on proximal raphe endings, the newly described *L.cribriareolata* sp. nov. can be easily distinguished from *L.madagascarensis* and *L.nosybeana*. Both Madagascar species share the same stria density (20–24 in 10 μm), which are denser than in *L.cribriareolata* sp. nov. which has 14–16 striae in 10 μm. Also *L.cribriareolata* sp. nov. has deeply positioned cribra which are not observed in either *L.madagascarensis* or in *L.nosybeana* (Bąk et al. 2019).

The presence of areola occluded with cribra in *Luticolacribriareolata* sp. nov. is a very rarely observed character in *Luticola*. Up until now only 3 cribrum-bearing *Luticola* species are known, *L.mutica* from Europe, *L.rionegrensis* from Rio Negro in South America and *L.subcrozetensis* from Maritime Antarctica (Levkov et al. 2013; Zidarova et al. 2016). From these taxa only *L.mutica* and *L.rionegrensis* have (same as *L.cribriareolata* sp. nov.) cribra positioned deeply within areola, while cribra in *L.subcrozetensis* are located almost at the external surface of the valve (Zidarova et al. 2016: 205, fig. 19). In contrast to cribra-bearing *Luticola* species (as well as all described *Luticola*), the internal hymen of *L.cribriareolata* sp. nov. does not form a regular continuous strip but forms strips of irregularly-shaped occlusions conjoined with occlusions of a longitudinal channel.

The newly described *L.halongiana* sp. nov. possesses slightly hooked proximal raphe endings with only small depressions on the stigma-bearing side. This species also shows highly variable shape of the valve apices. In contrast to other brackish-water *Luticola* with rhombic-elliptic or rhombic-linear margins, this species has a relatively broad central area. Also unique among brackish-water *Luticola* is the small, rounded stigma positioned almost midway between the valve center and valve margin. A similar position of the stigma is found in *L.madagascarensis*. However, *L.madagascarensis* can be distinguished from species described herein, based on their external proximal raphe endings with distinctive long grooves on the side opposite the stigma.

Also *Luticolamutica* (Kützing) D.G.Mann shows some similarities to *L.halongiana* sp. nov., however, it can be easily distinguished based on its narrower central area, less dense striae (16–18 vs. 20–24 in 10 µm) and presence of cribrum in areolae which does not occur in *L.halongiana* sp. nov.

### Biogeography of the *Luticola* studied

The biogeography of most of the established species has been originally observed and described from Indonesian Islands (Hustedt 1942, 1955), assigned to *Navicula* and later transferred to *Luticola* either in Round et al. (1990) or in Levkov et al. (2013). The latter species seems to have wide environmental amplitude as it can be abundant in brackish and freshwater habitats (this study; Levkov et al. 2013).

We saw great variability in the distribution and relative abundances. For example, *L.celebesica* and *L.belawanensis* were observed only rarely and found from one sample site. *L.inserata*, *L.seposita* and *L.tropica* occurred in high relative abundance and from a few sampling sites. The published data on their geographic distribution shows that some of them, e.g. *L.tropica*, are globally distributed in tropical estuaries and marine coasts (Fernandes et al. 1990; Navarro and Lobban 2009; Levkov et al. 2013; Straube et al. 2017; Glushchenko et al. 2017), whereas the others occur in more restricted areas like in waters from SE Asia to the coast of Australia (*L.belawanensis* and *L.inserata*). *Luticolacelebesica* was described from Makassar on Sulawesi Island, however, the species description does not indicate the habitat in which this species was found (Levkov et al. 2013). In the analyzed materials, this species occurred very rarely, which made it impossible to make a detailed description of the observed population which would include the ultrastructure of the valves. Several specimens of *L.celebesica* have been observed only on the mud flat of Rú Chá Mangrove near Hue, the western South China Sea coast in Central Vietnam. *Luticolabelawanensis* was described for the first time from the mouth of the River Belawan on Sumatra and later it was reported from mangrove forests in Vietnam by Levkov et al. (2013) and Glushchenko et al. (2017). We observed *L.belawanensis* in periphyton from oysters shell in offshore aquaculture areas, Quáng Yên, in Ha Long region of NE Vietnam from Western South China Sea. All reports of these two taxa confirm that both of them prefer brackish-water conditions.

Likewise, *Luticolainserata* was described from the Sumatran coast at the mouth of the Belawan River (Hustedt 1942, 1955; Simonsen 1987). The species has a very rich published record of occurrence from the coastal waters of SE Asia (Indonesia, Vietnam) (Hustedt 1955; Amossé 1969; Glushchenko et al. 2017) and NE Australia (Foged 1978). Levkov at al. (2013) characterize *L.inserata* as a tropical, brackish-water species. This species shows great variability of shape, from lanceolate-elliptical in early stages of life cycle (Fig. 8A, B; Glushchenko et al. 2017) to broadly elliptic in smaller specimens (Fig. 8C–R). We have observed it in high relative abundance in sample SZCZ26472 from periphyton from oysters shell in offshore aquaculture from Quáng Yên, Western South China Sea, in Halong region of NE Vietnam. Despite it being a commonly reported species, a detailed description of valve ultrastructure of this species was not published until the observations presented herein. We have observed it in high relative abundance in sample SZCZ26472 and have been able to resolve the valve ultrastructure. In our SEM observations this species shows some unique characters including areola (both on valve face and valve mantle) with short spines on margins and only partially elevated raphe sternum. Both of these features allow it to be distinguished from *L.seposita* which has an almost identical valve outline and overlapping valve size dimensions (Table 2).

*Luticolaseposita* was described by Hustedt (1942) from Mahalon-See (Lake Danau Mahalona) on Sulawesi Island. The species was considered to prefer nutrient poor, circumneutral waters with elevated metal concentration and up until now was not reported from marine habitats. In our study *L.seposita* was only observed in an epilithic sample from Fenjiezhou island on the coast of Hainan Island, NW South China Sea in high relative abundance. Seemingly, *L.seposita* is capable of adapting to a broad array of environmental conditions (Levkov et al. 2013, this study). It’s also worth mentioning that *L.seposita* was reported from Australia but the valves shown here (John 2020: fig. 141O–Q, p. 117) have a much larger central area that is bordered by a higher number of shortened striae. It is highly possible that these Australian specimens do not represent *L.seposita*, but another (possibly) undescribed species.

*Luticolatropica* is reported in the diatomological literature as a widely distributed species confined to tropical estuaries and marine coasts. This species is based on Naviculainseratavar.undulata Hustedt (Hustedt 1955) and its type habitat is the mouth of Belawan River on the Sumatran coast. The species has been reported from marine coasts and estuaries of the Atlantic Ocean in Brazil (Fernandes et al. 1990), East African coast of Natal in South Africa (Cholnoky 1960), Pacific Ocean tropical Islands (Navarro and Lobban 2009) and tropical coasts of Ha Long Bay in NE Vietnam and Hainan Island (this study). From all reported taxa only *Luticolatropica* seems to have the widest (pantropic) distribution among the brackish-water and marine taxa treated here. Until now, it has been reported from the mouth of rivers and coastal waters of South-East Asia (Vietnam), Africa (Ghana, Gambia, KwaZulu-Natal), South America (Brazil) and the Pacific tropical island of Guam (Cholnoky 1960; Foged 1966, 1986; Navarro and Lobban 2009; Levkov et al. 2013; Straube et al. 2017). Despite its wide distribution, particular populations do not show significant morphological differences (Table 2).

From eight identified taxa (including three new to science), seven of them were found only in samples collected from marine ecosystems (salinity 22.0–32.8 psu). Based on the literature data as well as on the presented results, it seems that all of them are species that find their ecological optimum in marine habitats. Only *L.celebesica*, which was described from Sulawesi Island (Indonesia), seems to be a species that prefers waters with increased salinity (brackish environment) and does not occur in typically marine diatom assemblages.

## Supplementary Material

XML Treatment for Luticola orientalis

XML Treatment for Luticola cribriareolata

XML Treatment for Luticola halongiana

XML Treatment for Luticola belawanensis

XML Treatment for Luticola celebesica

XML Treatment for Luticola inserata

XML Treatment for Luticola seposita

XML Treatment for Luticola tropica
